# Complementary and alternative medicine: knowledge, interest and attitudes of medical students

**DOI:** 10.1186/1753-6561-6-S4-P29

**Published:** 2012-07-09

**Authors:** KP Loh, H Ghorab, E Clarke, R Conroy, J Barlow

**Affiliations:** 1 Royal College of Surgeons in Ireland, Ireland; 2 Division of Population Health Science, Royal College of Surgeons in Ireland, Ireland; 3 Department of Pharmaceutical & Medicinal Chemistry, Royal College of Surgeons in Ireland, Ireland

## Introduction

Complementary and alternative medicine (CAM) is a growing industry in the healthcare system. It has been reported that medical students across the globe have an interest in learning about CAM in addition to conventional medicine. In Ireland, to date there has not been a study that evaluates the knowledge of, interest in and attitude of Irish medical students towards CAM. This research can serve as a pilot study to inform Irish medical schools on the need to introduce CAM into the medical curriculum.

## Methods

The survey instrument was a modified design based on previously published studies carried out in other geographical areas. All medical students within the undergraduate and graduate entry (GEP) programmes at the Royal College of Surgeons in Ireland were invited to participate in the study. SPSS software was used to analyse the results of the questionnaires.

## Results

The survey completion rate was 20.1%. A majority of students (78.4%) thought that CAM knowledge is important for their future career as physicians. Approximately 65% of students reported that they have not acquired sufficient knowledge about CAM from medical school, and 50.2% of students believe CAM should be incorporated into the medical curriculum. Pre-clinical years (49.4%) were suggested as the most appropriate time to learn about CAM, and lectures were rated as the best teaching method (46.0%). Knowledge of CAM modalities was generally rated as minimal or none by students. Among the 15 CAM modalities incorporated in the survey, massage, acupuncture and meditation received the highest interest from students. Students who believe in a religion had a higher interest in CAM (P<0.05). In terms of their personal view, massage, spirituality and acupuncture received the highest positive responses. Attitudes towards CAM were positive from students. Lower willingness to use CAM was seen in clinical students (p<0.05).

## Conclusions

It is important for the faculty of Irish medical schools to consider the possibility of integrating CAM education into the conventional medical curriculum in a systematic manner to better prepare students in their future career.

